# Pneumoperitoneum in a Micropremie: Not Always NEC

**DOI:** 10.1155/2012/295657

**Published:** 2012-04-03

**Authors:** Ahmed Khan, Koert de Waal

**Affiliations:** Neonatal Intensive Care Unit, John Hunter Children's Hospital, New Lambton, Newcastle, NSW 2305, Australia

## Abstract

Pneumoperitoneum in the newborn is an acute surgical emergency requiring immediate surgical intervention to ensure survival. It refers to radiological evidence of rupture of an air-containing viscus with resultant soiling of the peritoneal cavity. A female baby was born preterm at 29 weeks with birth weight of 650 grams. She developed abdominal distension on day 6, and abdominal radiography revealed presence of free air in the peritoneum. She proceeded for a laparotomy, and intraoperative findings revealed blood in the peritoneum with an area of inflammation and a small perforation. About 5 cm of the inflamed bowel was resected, and an end to end anastomosis performed. The histopathology of the specimen was consistent with Meckel's diverticulum. Symptomatic Meckel's diverticulum is usually seen in the first two years of life, and perforation is a rare presentation. Perforated Meckel's diverticulum in a premature newborn is very rare, and a review of literature reveals only one other reported case. Newborn Meckel's perforation cases often mimic necrotizing enterocolitis, although many present without any feature of peritonitis. Establishing a preoperative diagnosis of perforated Meckel's is difficult and may not be essential as the treatment remains the same. However, prompt surgical intervention confers a good prognosis in neonates with isolated perforated Meckel's diverticulum.

## 1. Introduction

Meckel's diverticulum (MD) is the commonest congenital abnormality of the small intestine with prevalence of 2 percent in the general population. Most complications manifest in childhood, usually beyond the age of 4 months [1]. It is uncommon in newborns and rare in the preterm population. It can present in neonates as bowel obstruction and pneumoperitoneum. Bleeding per rectum is uncommon in neonates unlike in the paediatric population [2]. We report a severely growth-restricted preterm presenting with a perforated MD.

## 2. Case Report

A live female baby was born at 29-week gestation with a birth weight of 650 grams. The mother was a 30-year old G2P0, and her serology was negative. She was a smoker and had abnormal Doppler flows in the umbilical vessels. The antenatal scans revealed intrauterine growth restriction (IUGR), and she received a full course of antenatal steroids at 28 weeks. She was hospitalised for reduced foetal movements and proceeded to deliver by an emergency LSCS for a pathological CTG. She was born with Apgar scores of 6 and 9. She received surfactant and was extubated to CPAP. She had a symptomatic patent ductus arteriosus on day 3 of life requiring inotropic support with dopamine and had successful closure following treatment with a 3 doses of IV ibuprofen. On day 6 of life she had a clinical deterioration with a distended abdomen. An abdominal X-ray revealed presence of free air in the peritoneum, and she proceeded to theatre for a laparotomy on suspicion of a gut perforation. Intraop findings revealed blood from the stomach to the midileum with a large area of inflammation and perforation. About 5 cm of the inflamed bowel was resected, and an end to end anastomosis performed. The histopathology of the specimen was consistent with Meckel's diverticulum. She recovered well from this surgery but demised following a suspected aspiration episode 5 weeks later.

## 3. Discussion

Symptomatic Meckel's diverticulum usually occurs in the first two years of life. A perforation of the Meckel's diverticulum is less common and rare in the neonatal period. Diverticular length and base diameter are well known predisposing factors to complications, and long, narrow-based diverticula are thought to be more prone to obstruction or inflammation [3]. Bleeding per rectum is uncommon in neonates, and only one reported case was found in the literature [2]. A review of literature of 8 cases found four (50%) of neonates presented with symptoms of bowel obstruction, three (38%) presented with pneumoperitoneum, and one (12%) with *E. coli* sepsis. The median age at presentation was 4.5 (±4.3) days [4].

 Pneumoperitoneum in the newborn is an acute surgical emergency with serious implications requiring immediate surgical intervention to ensure survival. The term “pneumoperitoneum” refers to radiological evidence of rupture of an air-containing viscus with resultant gross soiling of the peritoneal cavity [5]. Very few cases of perforated Meckel's are described in neonatal literature. These cases often mimic necrotizing enterocolitis although many are without any features of peritonitis [2]. Perforation of Meckel's diverticulum is commonly due to underlying pathology, but spontaneous perforation has been reported in literature [6].

A review of literature reveals only one other reported case of perforated Meckel's diverticulum in a micropremie. That infant had a significant number of factors that may have predisposed him to intestinal perforation, including antenatal and postnatal steroid therapy, hypoxia, and poor intrauterine blood flow [4]. Our patient had a similar profile but had received IV ibuprofen for treatment of a symptomatic patent ductus arteriosus instead of postnatal steroids.

## 4. Conclusion

Perforated Meckel's diverticulum in a premature newborn is very rare and can present with a pneumoperitoneum. Establishing a preoperative diagnosis of perforated Meckel's diverticulum is difficult and is not essential as the treatment remains the same, which is a surgical laparotomy. Prompt surgical intervention usually confers a good prognosis and intact survival in neonates with isolated perforated Meckel's diverticulum.
